# Nanomedicines against Chagas disease: a critical review

**DOI:** 10.3762/bjnano.15.30

**Published:** 2024-03-27

**Authors:** Maria Jose Morilla, Kajal Ghosal, Eder Lilia Romero

**Affiliations:** 1 Nanomedicine Research and Development Centre (NARD), Science and Technology Department, National University of Quilmes, Roque Sáenz Peña 352, Bernal, Buenos Aires, Argentina; 2 Department of Pharmaceutical Technology, Jadavpur University, 188, Raja Subodh Chandra Mallick Rd., Jadavpur, Kolkata 700032, West Bengal, India

**Keywords:** benznidazole, liposomes, nanocrystals, nanomedicines, nanoparticles, *Trypanosoma cruzi*

## Abstract

Chagas disease (CD) is the most important endemic parasitosis in South America and represents a great socioeconomic burden for the chronically ill and their families. The only currently available treatment against CD is based on the oral administration of benznidazole, an agent, developed in 1971, of controversial effectiveness on chronically ill patients and toxic to adults. So far, conventional pharmacological approaches have failed to offer more effective and less toxic alternatives to benznidazole. Nanomedicines reduce toxicity and increase the effectiveness of current oncological therapies. Could nanomedicines improve the treatment of the neglected CD? This question will be addressed in this review, first by critically discussing selected reports on the performance of benznidazole and other molecules formulated as nanomedicines in *in vitro* and *in vivo* CD models. Taking into consideration the developmental barriers for nanomedicines and the degree of current technical preclinical efforts, a prospect of developing nanomedicines against CD will be provided. Not surprisingly, we conclude that structurally simpler formulations with minimal production cost, such as oral nanocrystals and/or parenteral nano-immunostimulants, have the highest chances of making it to the market to treat CD. Nonetheless, substantive political and economic decisions, key to facing technological challenges, are still required regarding a realistic use of nanomedicines effective against CD.

## Introduction

Nanomedicines are used to solve the problems posed by poor solubility and/or permeability and high toxicity of drugs with low molecular weight [1–2]. Different 2-nitroimidazole-based nanomedicines against Chagas disease (CD) to reduce the toxicity and increase the effectiveness of benznidazole (BNZ) treatment have been preclinically screened in the last two decades (see the recently reviewed BNZ-based preclinical anti-CD nanomedicines [3]). But how realistic is thinking of nanomedicines to treat CD? To answer this elemental question, selected preclinical reports will be thoroughly discussed in this review. Then, by addressing current contexts and directions of nanomedical advances, the idea of using nanomedicines against CD will be critically analyzed.

## Review

### Chagas disease, a threat no longer limited to developing countries

Chagas disease is a parasitic, systemic, chronic, and often fatal infection caused by the protozoan *Trypanosoma cruzi* [4]. The World Health Organization classifies CD as the most prevalent of poverty-promoting neglected tropical diseases, and the most important parasitic one. Also known as American trypanosomiasis, CD is the third most infectious disease in Latin America; it is endemic in 21 countries and constitutes a global public health issue affecting six to eight million people [5]. Globally, CD creates an annual burden exceeding 800,000 disability-adjusted life years and $600,000,000 in healthcare costs [6]. Classically, the infectious cycle in the human host begins as an acute phase, asymptomatic except in children, where trypomastigotes circulate in the blood and intracellular amastigotes are usually found in hepatic macrophages. Amastigotes multiply and differentiate into trypomastigotes, which are released back to the blood after cell rupture. The acute phase is followed by an indeterminate, asymptomatic phase. Ten to thirty years after the acute phase, 30%–40% of patients will develop a chronic phase. This phase presents typical denervation and fibrosis of cardiac or digestive muscles, with scarcer intracellular forms. The subsequent cardiac arrhythmias or progressive heart failure and sudden death are the highest attributable cost of the disease [7–8]. With 75 million people at risk, 30,000 new cases each year, and 12,000 deaths in 2019, less than 30% of infected people are diagnosed [5]. Currently, because of emigration, CD is becoming increasingly important in North America, Europe, Japan, and Australia [9–10]. Nearly 300,000 individuals in the United States are calculated to have CD, with up to 45,000 having cardiomyopathies [11].

The infection is treated with benznidazole, first manufactured by Roche (Roche 7-501, Rochagan, *N*-benzyl-2-(2-nitro-1*H*-imidazol-1-yl)acetamide). BNZ is currently available in the United States after being approved by the US Food and Drug Administration in 2018. Since the early 70s, patients received the same BNZ-based treatment, which is long, toxic to adults, effective in recently infected people, and controversially effective in the chronic phase [12]. A recommended course of 5–10 mg BNZ/kg orally, is divided into two daily doses for 60 days after meals [13].

### On BNZ metabolization, doses, and toxicity

BNZ is a prodrug that requires activation by oxygen-insensitive NADH-dependent trypanosomal type-I nitroreductase (NTRI), found in some protozoan parasites, but not in humans [14]. This activation produces hydroxy and hydroxylamine intermediates in a two-step, two-electron transfer reaction, culminating in 4,5-dihydro-4,5-dihydroxyimidazole, whose breakdown releases the reactive dialdehyde glyoxal, which, in the presence of guanosine, generates guanosine–glyoxal adducts. These reactive metabolites are toxic to the parasite, resulting in its fast killing. Lately, it was suggested that the major metabolic impact of BNZ is on the glutathione (and trypanothione) pathway so that covalent binding of BNZ with low-molecular-weight thiols and with protein thiols is the drug’s primary mode of action against *T. cruzi* [15].

In mammalian cells, BNZ is reduced by oxygen-sensitive nitroreductases. During its anaerobic nitro reduction, primarily in the hepatic microsomal fraction, BNZ generates reactive metabolites that bind to the host’s DNA, proteins, and lipids. The nitro reduction also occurs in fecal matter, with an intensity that increases with age. The toxicity of these products on rodent adrenals, colon, and esophagus has been extensively studied by Castro’s group in Argentina. The same group reported that BNZ also inhibits the metabolism of several xenobiotics transformed by the cytochrome P450 system and their metabolites react with fetal components *in vivo* [16–18]. The consumption of glutathione resulting from its reaction with BNZ metabolites would later lead to oxidative stress processes. One of the main disadvantages of BNZ pharmacotherapy is the high doses administered, thought to be responsible for the pronounced idiosyncratic adverse drug reactions (ADRs), caused by BNZ reduction products, which are maximal in adults and lead to treatment discontinuation [19]. Typical ADRs include headache, anorexia, weakness and/or lack of energy, skin rash, gastrointestinal complaints, and mild, peripheral neurological effects [20].

The dosage of BNZ has been reported to be inadequate [21]. In children, markedly lower plasma BNZ concentrations than those previously reported in adults treated with comparable BNZ milligram per kilogram doses (possibly due to a higher clearance/bioavailability), but still retaining a high therapeutic response, were detected [22]. This finding led to the assumption that in adults the BNZ treatment could be overdosed. Unlike adults, children show few ADRs; therefore, the existence of a potential direct correlation between drug concentration and the incidence of ADRs was suggested. Data from simulations showed that reducing the cumulative dose from 2.5 mg/kg/12 h to 2.5 mg/kg/24 h rendered BNZ plasma concentrations within the accepted therapeutic range of 3 to 6 mg/L [22]. This is an important point: When BNZ toxicity is attributed to overdosing, it could be simply reduced by reducing its dose. The implementation of shorter treatments has also been proposed [23–24].

Other reports suggest, nonetheless, that the overdosage of BNZ as the origin of ADRs is debatable. Studies implemented with adult patients not only failed to connect the manifestation of ADRs with the BNZ plasma levels but also opened the theory of genetic background or immunological profile in addition to hypersensitivity reactions to BNZ [25–26]. Accordingly, reducing BNZ doses to decrease its plasma levels would not have an impact on its toxicity [27].

### Oral BNZ-based nanomedicines aimed to increase BNZ solubility

Besides uncertainties about BNZ dose, its biopharmaceutical classification differs according to the data source and interpretation. In the early 1980s, BNZ was reported to be readily absorbed, highly lipophilic, and extensively metabolized, with only 5% of the dose excreted unchanged in the urine [28]. Since poorly soluble drugs are those whose solubility is below 1 mg/mL over the physiological pH range and the BNZ solubility in distilled water or simulated gastric and enteric fluids oscillates between 0.2 and 0.4 mg/mL [29–30], BNZ is considered a poorly soluble drug. Some authors classify BNZ as a class-II drug, according to the Biopharmaceutical Classification System (BCS) [31], which means that it is a poorly soluble but permeable molecule. The limitation in the absorption of class-II drugs is due to their rate of dissolution, except at very high doses. Their bioavailability is variable but can be increased by augmenting their dissolution rate, and *in vitro*–*in vivo* correlation is normally applied [31]. These drugs are suitable for sustained release and controlled release formulations that provide more stable and predictable plasma levels. Drug solubility can be increased by employing strategies from classical pharmaceutical technology such as lyophilization, micrometerization, microemulsion, the inclusion of surfactants, solid dispersion, and the use of complexing agents such as cyclodextrins, Zer-Os tablet innovation, soft gels, and triglas [32]. Nanomedicines can also be used to increase the oral bioavailability of BNZ, and their contribution is examined below.

The *in vitro* performance of BNZ loaded into nanoparticles (Nps) is shown in Table 1. In many of these reports, formulations were tested on different parasite stages (epimastigote, trypomastigote, and amastigote), and their cytotoxicity was assessed on mammalian cells [33–38]. However, orally administered nanomedicines do not cross the intact gastrointestinal epithelium and would never be uptaken by target cells, except enterocytes. During gastrointestinal transit, biodegradable nanoparticles are degraded or not absorbed, leaving only released BNZ available for absorption [39–40]. Other studies determined the release profile of BNZ in different media [41–44] and its permeability across Caco-2 cells [43–44].

**Table 1:** *In vitro* performance of BNZ-based nanomedicines.^a^

Type of nanomedicine/ composition	Physicochemical properties	*In vitro* assays	Outcome	Ref.

vesicles, SLN, NLC and cyclodextrins (CDs)	vesicles: ≈200 nm CDs: 5–10 μm SLN: ≈170 nm, −21 mV ζ-potential NLC: ≈200 nm, −26 mV ζ-potential	cytotoxicity on L-929 cells and HepG2 cells activity on epimastiotes, trypomastigotes, and amastigotes of CL strain, clone B5	SLN less active than BNZ, NLC less active and more toxic than CD vesicles low EE CDs best-balanced anti-trypanosoma activity /toxicity, (less cytotoxicity of BNZ-CDs than BNZ without reduction of trypanocidal activity)	[33]
CaCO_3_ Nps	42 ± 8 nm	cytotoxicity on LLC-MK2 cells activity on epimastigotes, trypomastigotes, and amastigote of Y strain	less toxicity and higher selectivity, with anti-trypanosoma activity at 25 times lower concentrations of BNZ	[34]
mesoporous silica Nps (MCM-41) chitosan succinate covalently attached	−11.5 ± 0.5 mV ζ-potential	activity on epimastigotes of CL Brener strain	the same anti-trypanosomal effect as that of BNZ-MCM-41 at 30 times lower BNZ concentration	[35]
Nps based on Eudragit^®^ RS PO and Eudragit^®^ RL PO	200–300 nm; 24–36 mV ζ-potential; EE: 78% DL: 18% w/w	*in vitro* release in 0.1 N HCl (pH 1.2)	increased dissolution rate of drug from Nps	[41]
zeolitic imidazolate framework ZIF-8 (BNZ@ZIF-8)		*in vitro* release at pH 4.5 and 7.6	at pH 4.5, BNZ@ZIF-8 showed a faster release with a burst effect, while, at pH 7.6, it showed prolonged and controlled release	[42]
nanocrystals		*in vitro* release in FaSSGF, FeSSIF, and FaSSIF integrity of tight junction dynamics and permeability on Caco-2	safety and increased permeation through the Caco-2 cells with minimal interactions with mucin glycoproteins	[43]
lipid nanocapsules Lipoid S 100, Kolliphor^®^ HS 15 and Labrafac^®^ WL 1349 oil phase at three oil/surfactant ratios	30, 50, and 100 nm; PDI < 0.07; −1.59 to −0.96 mV ζ-potential	release in FaSSGF, FeSSIF, and FaSSIF with pancreatic enzymes permeability on Caco-2	NCPs protected BNZ in simulated gastric fluid and provided sustained release in a simulated intestinal fluid improved BNZ permeability	[44]
NLC myristyl myristate/crodamol oil/poloxamer 188	150 nm; −13 mV ζ-potential; EE: 80%	release at pH 6.8 haemolysis cytotoxicity on CHO and Vero cells activity on trypomastigotes and amastigotes of strain K98	biphasic drug release profile with an initial burst release followed by a prolonged phase trypanocidal activity similar to that of free BNZ, with lower cytotoxicity to mammalian cells	[36]
NLC compritol, crodamol, Tween 80 and poloxamer 407 (P407)	110 nm; PDI: 0.19 −18 mV ζ-potential; EE: 83%; DL: 1.64	haemolysis cytotoxicity on L929 cells activity on epimastigotes of Colombian strain	NLC-BNZ had higher trypanocidal activity than free BNZ with low cytotoxicity to mammalian cells	[38]
Polymeric Nps cashew phthalate gum		activity on epimastigotes and trypomastigotes cytotoxicity on macrophages	Nps enhanced trypanocidal activity, and reduced cytotoxicity	[37]

^a^Abbreviations: DL – drug loading; EE – encapsulation efficiency; FaSSGF – fasted-state simulated gastric fluid; FaSSIF – fasted-state simulated intestinal fluid; FeSSIF – fed-state simulated intestinal fluid; NLC – nanostructured lipid carriers; PDI – polydispersity index; SLN – solid lipid nanoparticles.

Between 2012 and 2018 the BERENICE (BEnznidazol and triazol REsearch group for Nanomedicine and Innovation on Chagas diseasE) consortium, aiming for a new, cheaper, more effective, better-tolerated solutions for chronic Chagas patients, was constituted [45]. The consortium, financed by the Seventh Framework Programme, was coordinated by the Institut Catala De La Salut and included ten researchers from Spain, endemic countries’ institutions (Brazil and Argentina), and private pharmaceutical companies. The project started proposing a sublingual formulation of BNZ within liposomes or lipid nanoparticles, assuming the intact formulations could reach the blood, avoid the hepatic first-pass metabolism, and reduce the toxicity of BNZ. The project, however, failed in its attempt to incorporate BNZ into liposomes, while lipid nanoparticles could not be formulated into sublingual tablets. The project changed to formulate BNZ/hydroxypropyl-β-cyclodextrin complexes. These complexes were prepared on a scale seven times larger than in the laboratory and showed a comparable *in vitro* activity to free BNZ [33]. Formulated as oral tablets containing a reduced dose of BNZ/cyclodextrin (50% loading of BNZ (% BNZ/total mass)) and administered to a murine model, BNZ/cyclodextrin did not overcome the efficacy of free BNZ during the acute phase of the infection. The project eventually gave up on its attempts to formulate BNZ in nanomedicines and focused on clinical trials of reduced BNZ doses for the treatment of the chronic phase [23].

In parallel to the BERENICE project, several reports showed the *in vivo* performance of different nanomedicines capable of increasing the solubility of BNZ (Table 2). Trypomastigotes are known to display high resistance to the trypanocidal effect of BNZ [46–47]; replicative epimastigote and amastigote forms of the parasite, instead, would be sensitive to cumulative doses of BNZ. Arguing that a reduction in the cumulative dose of BNZ would reduce its toxicity without losing effectiveness [48], BNZ has recently been formulated as nanocrystals (NCs). The solubility of BNZ formulated as nanocrystals prepared by nanoprecipitation using the non-ionic surfactant poloxamer 188 as a stabilizer (BNZ-NC) was increased 10-fold (from 0.4 mg/mL for bulk BNZ to 3.99 mg/mL for BNZ-NC) [49]. After oral administration of BNZ-NC to an acute murine model infected with the Nicaragua strain of *T. cruzi* at 300 mg/kg total dose (TD) (30 days treatment) and 375 mg/kg TD (15 days treatment), which was about half of the 750 mg/kg free BNZ TD administered at 50 mg/kg/day over 15 days, both BNZ-NC and free BNZ showed 100% survival at 50 days post-infection (dpi) [50]. In a model of immunosuppressed chronic phase, 750 mg/kg BNZ-NC TD showed the same efficacy as 1500 mg/kg free BNZ. Immunosuppressed mice treated with BNZ-NC exhibited 40% of PCR negative samples; 50% of the mice showed negative IgG titers after 3 months, and 100% after 6 months. In contrast, parasites were detected in blood from mice treated with free BNZ, and *T. cruzi* antibodies were detected for up to 6 months. BNZ-NC decreases parasite burden, heart inflammation, and lesions [49]. The intermittent administration of BNZ-NC at 75 mg/kg (975 mg/kg TD) to a chronic murine model was also tested; it was as equally effective (parasite load, *T. cruzi*-specific antibodies levels, degree of fibrosis, frequency of IFN-γ producing cells, and improvement of electrocardiographic alteration) as intermittent free BNZ at 100 mg/kg (1300 mg/kg TD). BNZ-NC induced a 57% reduction in cardiac inflammation but failed to overcome the more significant reduction provided with intermittent free BNZ [51]. Overall, employing intermittent and lower cumulative doses of BNZ-NC, the authors showed comparable therapeutic effects to conventional treatment with free BNZ. These studies, however, did not compare the plasma levels of BNZ resulting from administering nanocrystals to rodents with identical doses of free BNZ.

**Table 2:** *In vivo* performance of oral BNZ-based nanomedicines.^a^

Type nanomedicine/ composition	Physicochemical properties	*In vivo* assays	Outcome	Ref.

nanocrystals (BNZ-NC) BNZ dispersed in poloxamer 188	63.3 ± 2.82 nm; PDI: 3.35 ± 0.1; −18.30 ± 1.0 mV ζ-potential BNZ-NC dispersed in olive oil for administration	acute model, C3H/HeN mice, Nicaragua strain BNZ: 50 mg/kg/day for 15 days (750 mg/kg TD) BNZ-NC: 50, 25, and 10 mg/kg/day for 30 days (1500, 750, and 300 mg/kg TD, respectively) and 50 and 25 mg/kg/day for 15 days (750 and 375 mg/kg TD, respectively)	without treatment 15% survival at 50 dpi with BNZ and BNZ-NC 100% survival	[50]
BNZ-NC same formulation as in [50]		acute model, C3H/HeN mice, Nicaragua strain infected + immunosuppression (60 dpi) BNZ: 50 mg/kg/day for 30 days (1500 mg/kg TD) BNZ-NC:10, 25, and 50 mg/kg/day for 30 days (300, 750, and 1500 mg/kg TD, respectively) starting 2 dpi.	without treatment 15% survival BNZ and BNZ-NC survived until 92 dpi	[49]
BNZ-NC same formulation as in [50]		chronic model, C57BL/6J mice, Nicaragua strain BNZ: 50 and 75 mg/kg/day for 30 days (1500 and 2250 mg/kg TD, respectively) or 13 times one dose every seven days (it) of 75 or 100 mg/kg (975 and 1300 mg/kg TD, respectively) BNZ-NC: 25 and 50 mg/kg/day for 30 days (750 and 1500 mg/kg TD, respectively) or 13 times one dose every seven days (it) of 50 and 75 mg/kg/day (650 and 975 mg/kg TD, respectively) starting 90 dpi	All infected mice survived (210 dpi). Untreated mice median parasite load 8.9 Eq/mL BNZ (75 mg/kg × 30 days) and BNZ (it) 100, showed 80% and 75% without parasitaemia, respectively BNZ-NC (it) 50, 80% negative qPCR no parasite load could be detected in any other BNZ-NC group	[51]
BNZ-SNEDDSs Miglyol^®^810N, Capryol 90^®^, Lipoid S75, Labrasol^®^, *N*-methyl pyrrolidone (30:15:20:15:20 v/v)	25 mg/mL BNZ; 500 nm	acute model, BALB/c mice, Y strain 100 mg/kg/day starting 4 dpi for 20 days	57% cure for both free-BNZ and BNZ-SEDDSs groups	[52]
BNZ-NFX-SNEDDSs Labrasol, Labrafil 1944CS, Capryol 90 50.00:10.12:39.88 (w/w)	132 ± 7 nm; PDI: 0.610 ± 0.056; 33.1 ± 2.4 mV ζ-potential	acute model, BALB/c mice, Y strain. NFX (50 mg/kg/day) or BNZ (50 mg/kg/day) orally daily for 5 days NFX-BNZ-SNEDDSs (25 and 50 mg/kg/day), BNZ-SNEDDSs (50 and 100 mg/kg/day) administered orally once a day for five consecutive days starting 5 dpi	without treatment 15–17.5 days survival NFX and BNZ increase survival to 30 dpi	[53]
NCP Eudragit L100 (0.25 g), BNZ (0.025 g), sorbitan monooleate (0.19 g), medium-chain triglycerides (413 μL), ethanol (67 mL), and aqueous phase (polysorbate 80 (0.19 g) and water))	146 ± 0.6 nm; PDI: 0.15 ± 0.01; −12.8 ± 0.87 mV ζ-potential; EE: 96%	acute model, Swiss mice, Y strain BNZ-NCP 5, 10, 15, and 20 mg/kg/day starting 2 dpi for eight days	BNZ (100 mg/kg/day) reduced parasitemia and 100% survival after 30 days	[54]

^a^Abbreviations: NCP – nanocapsules, IV – intravenous, it – intermittent.

Other authors have recently reported the formulation of BNZ into self-nanoemulsified drug delivery systems (SNEDDSs). SNEEDSs provide a pediatric liquid formulation of BNZ, which is only marketed as solid tablets. SNEDDSs are isotropic mixtures of oil, surfactants, and co-surfactants that form submicrometer-droplet emulsions under agitation in water or gastrointestinal fluids. BNZ-SNEDDSs resulted in the same percentage of cure (57%) as free BNZ in an acute murine model infected with the Y strain of *T. cruzi* [52]. In a subsequent report, BNZ and nifurtimox (NFX) were loaded in a solid formulation of SNEDDSs, and their administration at 25 and 50 mg/kg/day (BNZ and NFX, respectively) over 5 days ensured 30 dpi survival in two-thirds of treated animals [53]. BNZ was also loaded in Eudragit L-100 nanocapsules (BNZ-NCP). Their administration at 20 mg/kg/day for 8 days yielded reduced parasitemia, and 50% of treated mice survived 30 dpi [54].

### Intravenous BNZ-based nanomedicines

According to other authors, BNZ is a class-III drug, that is, a soluble and poorly permeable molecule. The classification is based on BNZ’s dose number (which for BNZ is 1; dose numbers ≤ 1 correspond to highly soluble drugs) and on its calculated partition coefficient value clogP, a lipophilicity indicator and the most critical parameter predictor of passive membrane permeability (which for BNZ is 0.9; a clogP value below 1.35 is indicative of low permeability) [29,55]. In contrast, others consider BNZ as a class-IV drug, this is, a poorly soluble and poorly permeable molecule [30] with low tissue distributions in healthy mice [56].

By loading into intravenously administered nanomedicines, the biodistribution of poorly permeable and poorly soluble drugs could be controlled, and their activity against selected targets improved. For BNZ, the avoidance of healthy tissues and the reduction of hepatic first-pass metabolism is expected to minimize its toxicity [57–58]. Except on immediately accessible targets such as epithelia, however, controlled biodistribution of nanomedicines requires intravenous injection [59–60]. A fraction of injected nanomedicines would passively accumulate in inflamed tissues and could be delivered to amastigotes after being endocytosed by infected cells. In addition, since endocytosis occurs with cellular energy expenditure, the internalization of BNZ would be independent of its permeability and dose. In this way, very small doses of BNZ could be site-specifically concentrated in areas of infection.

Intravenously administered nanomedicines can deliver minute drug amounts and mediate shorter, less toxic, and more effective treatments than conventional medicines. The effectivity of low liposomal amphotericin B doses used to treat lethal visceral leishmaniasis implemented in India in the mid-1990s is an excellent example [61–62]. Amphotericin B binds to parasite ergosterol precursors, such as lanosterol, disrupting the parasite membrane. Since protozoan trypanosomatids such as Leishmania and Trypanosoma present ergosterol as a component of their membranes [63], short doses of liposomal amphotericin B were expected to act effectively against CD. Unfortunately, the trials did not exceed the preclinical phase. Liposomal amphotericin B cleared blood trypomastigotes and improved survival but did not cure mice [64–65]. All animals treated with liposomal amphotericin B relapsed after immunosuppression with cyclophosphamide, or amastigotes remained in tissues of all mice, particularly in the heart and brain after treating a chronic model of infection with *T. cruzi* CL strain [66]. The failure of liposomal amphotericin B was likely because therapeutic targets in CD are less accessible than in leishmaniasis, where only macrophages are infected.

The first report on BNZ-based nanomedicines intravenously administered to rats and mice dates back to 2004 [67] with disappointing results. An intravenous bolus of 0.7% w/w BNZ/lipid multilamellar liposomes administered two times a week over three weeks, at 0.4 mg BNZ/kg (2.4 mg/kg TD), increased blood BNZ levels and caused a transient and threefold higher accumulation of BNZ in the liver, which was insufficient to defeat the infection of an acute murine model infected with *T. cruzi* RA strain.

More recently, BNZ was formulated into polymersomes of poly(ethylene glycol)-*block*-poly(propylene sulfide) (BNZ-PS, 114.3 ± 4.1 nm; PDI: 0.11 ± 0.02; 4.92 ± 1.93 mV ζ-potential; DL: 1%) [68]. Only two injections of BNZ-PS (3 mg/kg TD) were highly potent in treating *T. cruzi*-infected mice (acute model; BALB/c mice; Y strain; 466-fold lower dose than oral free BNZ with 1400 mg/kg TD), caused no detectable hepatotoxicity, and completely abrogated the weight loss. BNZ-PS, but not free BNZ, significantly reduced the number of parasites in the heart and the inflammation.

In 2005, ultra-low doses of pH-sensitive nanoliposomes of etanidazole (a soluble 2-nitroimidazole; 0.63 mg etanidazole/kg/day in nine total doses, three doses per week over three weeks: 5.67 mg etanidazole/kg TD) reduced trypomastigotes in blood of an acute murine model infected with *T. cruzi* RA strain [69], to the same extent as orally administered BNZ at a 353-fold higher dose (100 mg/kg/day over 20 days: 2000 mg/kg TD [52]).

The study of the effect of pH-sensitive liposomes for etanidazole delivery to CD models was discontinued, but along with BNZ-polymersomes both showed that ultralow doses of the antiparasitic drug could reduce infection and increase survival. Nonetheless, the efficacy of these few experiments is uncertain, since their effect on chronic and immunosuppressed models, as well as the potential toxicity, remain unknown.

### Non-approved drugs-based nanomedicines

The *in vivo* activity of non-approved drugs loaded into lipid and polymeric nanoparticles orally and intravenously administered has also been tested (Table 3). For example, oral solid lipid nanoparticles loaded with a poorly bioavailable lipophilic cyclic compound derived from dithiocarbazate, effectively reduced parasitemia, diminished inflammation and lesions of the liver and heart, and resulted in 100% survival of an acute murine model [70].

**Table 3:** *In vivo* performance of nanomedicines based on non-approved drugs.

Administration route/drug/type of nanomedicine	Composition/properties	*In vivo* studies	Ref.

oral H2bdtc^a^ SLN	Na taurodeoxycholate, stearic acid, soya lecithin, and H2bdtc (0.12, 0.95, 0.48, and 0.02% w/v, respectively) 127 ± 0.130 nm; PDI < 0.3; −56.1 ± 4.40 mV ζ-potential	acute model, Swiss mice, Y strain BNZ 1 mg/kg/day H2bdtc and H2bdtc-SLN 1.4 mg/kg/day starting 5 dpi oral for 10 days	[70]
IV LYC polymeric NCs	PLA-PEG NC: 1:1 PLA-PEG and Resomer 203 (1.2% w/v), Epikuron 170 (0.4% w/v), Miglyol 810N (2.5% v/v) 105.3 ± 2.3 nm; PDI < 0.3	acute model, Swiss mice, Y strain 4th dpi for up to 20 consecutive days at 2 mg/kg/day	[71]
oral LYC polymeric NCs	same formulations as in [71]	acute and chronic models, Swiss mice, Y strain acute: from 4 dpi for up to 20 consecutive days 5 mg/kg/day; BNZ 100 mg/kg/day chronic: stating on 90 dpi for 20 days 2 mg/kg/day; BNZ 50 mg/kg/day	[72]
oral LYC polymeric NCs	PLA-PEG NC 107 ± 8 nm; PDI < 0.3; −31 ± 8 mV ζ-potential	acute and chronic models, Swiss mice, VL-10 strain (100% resistant to BNZ and NFX). free LYC and LYC-PLA-PEG-NC 8 or 12 mg/kg/day by oral gavage from 9 dpi for acute and from 90 dpi for chronic administered for 20 days	[75]
oral BNZ + curcumin polymeric Nps	PLGA (50:50) Nps	chronic model, C57BL/6 mice, Brazil strain BNZ (25 mg/kg/day) + Np PLGA CUR (200 mg/kg/day) for 30 days from 60 dpi	[76]
oral curcumin nanodispersion	Theracurmin 10 w/w% of curcumin, 2% of other curcuminoids, 46% of glycerin, 4% of gum ghatti, and 38% of water 190 nm	acute model, Swiss mice, Colombian strain 30 mg/kg day theracurmin for 30 days	[79]
IV ursolic acid polymeric Nps	PLC and poloxamer 407 173.2 ± 7.28 nm; PDI 0.09 ± 0.03; −36 ± 3.34 mV ζ-potential	acute model, C57BL/6 mice, Y strain starting 48 h post-infection for 7 days	[80]

^a^H2bdtc: 5-hydroxy-3-methyl-5-phenylpyrazoline-1-(*S*-benzyl dithiocarbazate).

Either orally or intravenously administered to acute and chronic murine models, poly(ᴅ,ʟ-lactide)-*block*-polyethylene glycol nanocapsules loaded with lychnopholide (LYC-PLA-PEG NCs), a lipophilic sesquiterpene lactone isolated from *Lychnophora trichocarpha* of poor solubility, which is degraded at extreme pH values, showed improved efficacy against *T. cruzi* infection. The most relevant results in the acute model were that equal cure rates were obtained for oral LYC-PLA-PEG-NCs and BNZ (62.5%). In contrast, intravenous LYC-PLA-PEG NCs caused 100% [determined by parasitological, fresh blood examination, haemoculture, peripheric blood PCR, and serological (ELISA) methods] cure rate compared to 75% for BNZ, while free LYC reduced parasitemia and improved mice survival but did not lead to a cure [71]. The most relevant results in the chronic model were that oral LYC-PLA-PEG-NCs yielded a 55.6% cure rate vs 0% for BNZ and free LYC. Intravenous LYC-PLA-PEG-NCs yielded a 50.0% cure rate vs 0% for free LYC and BNZ [72]. Intravenous LYC-PLA-PEG NCs increased 16-fold the body exposure, 26-fold the plasma half-life, and reduced 17-fold the plasma clearance in comparison with free LYC [73], protecting the host against the cardiotoxicity of LYC [74]. Higher doses (12 mg/kg/day) of oral LYC-PLA-PEG-NCs cured 75% of animals in the acute phase and 88% of those in the chronic phase of murine models [75].

Orally administered to acute and chronic murine models, PLGA Nps (PLGA-CUR Nps) loaded with curcumin (the most active polyphenolic flavonoid constituent of Curcuma longa rhizomes with low bioavailability) and free BNZ, induced anti-inflammatory effects and cardiac protection. A low BNZ dose (750 mg/kg TD) plus PLGA-CUR Nps, reduced cardiac hypertrophy and parasite load, chronic inflammation, fibrosis, and the levels and activities of cardiopathogenic biomarker enzymes and cytokines/chemokines (IL-1β, TNF-α, IL-6, and CCL5), matrix metalloproteinases (MMP-2 and MMP-9), and inducible enzymes (cyclooxygenase and nitric oxide synthase) implicated in leukocyte recruitment and cardiac remodelling [76]. More recently, Theracurmin^®^ (a natural product of Theravalues, Tokyo, Japan, that enhances the curcumin bioavailability 30-fold compared with curcumin powder [77–78]) showed immunomodulatory (reduced CCL2 in cardiac tissue, IL-15 in cardiac and skeletal tissue, plasma creatine kinase, and tissue leukocyte infiltration) and trypanocidal effects (reduction of parasitemia) in an acute murine model [79]. A complementary use of Theracurmin^®^ with BNZ therapy is suggested.

Intravenous polycaprolactone Nps loaded with ursolic acid (UR-PCL), a natural pentacyclic triterpene of low bioavailability and poor aqueous solubility used as a dietary supplement, was found to reduce twofold parasitemia, compared with a 3.5-fold reduction of BNZ, in an acute murine model [80]. However, while BNZ caused liver toxicity, UR-PCL was not toxic to liver and kidney.

### Which diseases has nanomedicine focused on in the last 28 years?

There are currently between 50 [81] and 60 [82] nanomedicines on the market, and nearly 560 in clinical trials, most of them in clinical phase I (33%) and phase II (21%) [83]. 15% of marketed nanomedicines are antibody–drug conjugates, such as Loncastuximab tesirine, launched in 2021 to treat B-cell lymphoma [84]. Nearly 10% are polymer–drug/protein conjugates such as polyethylene glycol-ʟ-asparaginase (Calaspargase pegol, Asparlas), launched in 2019 in the USA to treat acute lymphoblastic leukemia [85]. Another 10% are protein-based nanoparticles including Abraxane, the first formulation based on protein nanotechnology launched in 2005 [86]. Nearly 10% are inorganic nanoparticles such as the radiosensitizer Hensify, which in 2019 obtained CE Mark approval in the European Union for the treatment of locally advanced soft tissue sarcoma. This category also includes cancer imaging and diagnosis such as the MRI imaging agent Resovist, carboxydextran-coated superparamagnetic iron oxide nanoparticles approved for liver contrast-enhanced MRI102 [87]. Another 10% are nanocrystals, such as Tricor (approved in 2004) or Triglide (approved in 2005), used to improve the bioavailability of the anti-hypercholesterolemic fenofibrate [88–89].

Polymeric nanoparticles and cell-derived vehicles such as exosomes have not entered the market yet because of issues regarding quality control, large-scale repeatable preparation, effectiveness, and safety [90].

The remaining nearly 45% are lipid-based nanoparticles, constituting the most prevalent category of nanomedicines accessible in the market [91]. These include uni- or multilamellar liposomes (vesicles formed by bilayers of amphiphilic lipids), and lipid nanoparticles. The introduction of new preparation techniques on the industrial scale, such as microfluidic devices, contributes to their successful clinical translation and reduces the production cost to relatively affordable prices [92–94].

The main proportion of lipid-based nanoparticles are liposomes. These are used for the delivery of antitumoral drugs with low molecular weight, such as Doxil^®^ (for delivery of doxorubicin) launched in 1995, DoceAqualip^®^ (for delivery of docetaxel, devoid of polysorbate-80 and ethanol) launched in 2014, Onivyde^®^ (for delivery of irinotecan) launched in 2015, and Vyxeos^®^ (for synchronous delivery of cytarabine and daunorubicin) launched in 2017. These liposomes act mainly by passive targeting mechanisms upon intravenous administration. Parenteral liposomes employing the DepoFoam technology are used in clinical analgesia, that is, DepoDur^TM^ and Expare^®^, approved in 2004 and 2018, respectively.

The above summary shows that until now, most nanomedicines have been marketed to solve two big problems, namely (i) the low bioavailability and/or (ii) the high toxicity of drugs with low molecular weight. The most representative examples of the first group of nanomedicines are nanocrystals, carrier-free colloidal systems in the nanometer range (100–1000 nm), with a theoretical drug loading of 100%. They consist of pure drugs, usually in a solid amorphous state, with a minimal quantity of surface-active agents for stabilization. Nanocrystals are superior to microsuspensions at increasing the oral bioavailability of class-II drugs with low solubility, or low or irregular bioavailability, and promoting adhesion to the gastrointestinal wall [95]. The small size of the crystals is associated with a large surface area, which increases interactions with the dissolving medium and accelerates the dissolution rate. The latest marketed nanocrystals are for intramuscular injection and provide long-time delivery of drugs such as paliperidone palmitate, an atypical antipsychotic, or antiretrovirals. Excluding the anti-fungal griseofulvin (not a nanocrystal, but a micrometer-sized crystal), and anti-retrovirals, the remaining drugs formulated in nanocrystals are used to treat noncommunicable diseases (anti-emetic, immunosuppressant, antiarrhythmic, anti-chronic pain, anti-angina, anti-inflammatory and anti-hypercholesterolemic agents, appetite stimulants, and bronchial dilatators). The other big group of nanomedicines [89] are liposomes aimed to reduce the toxicity of oncological drugs by changing their biodistribution and pharmacodynamics, requiring intravenous administration. The success rate from phase 1 to approval, of antitumor nanomedicines is 6%, compared with 3.4% for classical oncological drugs [96].

The newest nanomedicines not only improve the pharmacokinetics and safety profile of classical medicines but also display higher effectiveness [97].

This portfolio of liposomal nanomedicines is now broadening to include other than oncological drugs, such as those to prevent deadly infections or treat chronic diseases [81]. Nanocort for instance, is a novel liposomal platform for intravenous administration of prednisolone to patients with chronic inflammatory diseases, such as ulcerative colitis, inflammatory bowel diseases, rheumatoid arthritis, and multiple sclerosis, in phase-II/III clinical trials sponsored by Enceladus Pharmaceuticals BVTM [98].

Newly available nanomedicines are not limited to the delivery of small drugs. Several anti-infective nanoparticulate vaccines, most of them for non-viral gene delivery have recently hit the market. Examples are these constituted by lipid nanoparticles made of phospholipids, cholesterol, polyethylene glycol (PEG) lipids, and ionizable synthetic lipids (ALC-0315 from BioNTech-Pfizer and SM-102 from Moderna Therapeutics) for enhanced delivery of messenger RNA (mRNA) encoding the spike protein of the SARS-COV-2 virus to antigen-presenting cells [82]. These vaccines were approved by the FDA, Pfizer-BioNTech COVID-19 in 2021 [99] and Moderna COVID-19 Vaccine in 2022 [100], after the approval in 2018 of Onpattro (Patisiran) [101], the first gene therapy based on lipid nanoparticles containing RNA interference, for the treatment of hereditary transthyretin-mediated amyloidosis. Vaccines made of lipid-based nanoparticles for delivery of mRNA are currently being investigated to protect against other viral diseases such as Zika, influenza, human immunodeficiency virus (HIV), respiratory syncytial virus cytomegalovirus, and bacterial diseases such as tuberculosis [102]. Another example of nanoparticulate vaccine is the anti-malarial Mosquirix™ (RTS, S/AS01), recommended by the World Health Organization in four doses for children in 2021. Mosquirix™ employs a liposome-based adjuvant, AS01 (GlaxoSmithKline) [103] that contains 3-*O*-desacyl-monophosphoryl lipid A and QS-21, a water-soluble triterpene glycoside (saponin) [104]. Although the vaccine has low efficacy, it has considerable advantages regarding general health: Four doses of the vaccine would avoid 116,480 instances of malarial infection and 484 fatalities per 100,000 immunized children.

There are good reasons to predict a bright commercial future for the abovementioned groups of nanomedicines. Currently, the approval of anti-tumor nanomedicines and the recruitment of nanomedicines for clinical trials related to infectious diseases are gaining momentum [82]. Moreover, economic forecast reports predict an additional 12.8% growth by the year 2025 driven by the evolution of vaccines against COVID-19 based on nanomedicines and the projected huge global demand for these products [105]. However, the clinical translatability of nanomedicines is still complex. Consider, for instance, the liposomal formulation of the antifungal amphotericin B AmBisome^®^, with significantly lower nephrotoxic effects compared to amphotericin B deoxycholate and launched in 1990 [106–107]. Remarkably, despite being used to combat visceral leishmaniasis [62], AmBisome^®^ was not made to treat a neglected disease, but to fight systemic mycoses resulting from immunosuppression caused by oncological treatments [108]. The big picture shows that nanomedicines, specifically the drug delivery field, are (and probably will be) focused on diseases that exclude parasitic diseases, regardless of their socioeconomic burden. In the next two sections, we will examine the general and particular factors leading to this situation.

### General barriers to the development of nanomedicines

Typical challenges in pharmaceutical development result from low efficiency and high attrition rate [109]. Unfortunately, these challenges are magnified in nanomedicine development [1,82,110], and the reasons can be summarized as follows:

**Difficult scale-up, structural characterization, and conservation:** The structure of nanomedicines is much more complex than that of drugs with low molecular weight. Because of that, nanomedicines are considered as non-biological complex drugs (NBCDs). NBCDs present heterogeneous molecular nature, difficult to be precisely controlled, and cannot be fully characterized by physicochemical analysis; nanoparticles are not a mere excipient, but the entire complex is the active pharmaceutical ingredient [111]. The transition from small laboratory batch sizes to large industrial volumes is the most challenging step in nanomedicine product development [112]. Slight structural changes induced during the industrial-scale production may modify pharmacokinetics, biodistribution, and pharmacodynamics of nanomedicines and alter their therapeutic properties and toxic profile [113–114]. The industrial quality control is much more complex than that of conventional pharmaceuticals, focused mainly on the properties of the low-molecular-weight drug constituting the active pharmaceutical ingredient [115–117]. Given their structural complexity and high surface area, nanomedicines are highly susceptible to aggregation, hygroscopicity, contamination, phase transition, amorphous-to-crystalline transitions, and degradation. It is critical to maintain batch-to-batch reproducibility (in terms of mean size, polydispersity, ζ-potential, and drug loading) not only during large-scale manufacturing [118] but also during storage. These characteristics reduce the affordability of nanomedicines in developing and low-income countries [119–120].

**Changes in current pharmacological paradigms:** The four paradigms are (i) the choice of right administration route, (ii) the need for analytical techniques different from those used in classical pharmaceutical technology, (iii) new toxicological assays, and (iv) suitable animal models: (i) Most nanomedicines, except nanocrystals/nanosuspensions, should be injected into the blood circulation. This is not convenient for many patients compared to oral or other non-invasive routes [121]. (ii) Drugs with low molecular weight diffuse readily across biological barriers and their concentration in blood is in equilibrium and related to achievable target tissue levels. The blood concentration of drugs loaded in nanomedicines, instead, is not indicative of the drug’s bioavailability. Because of their huge size, nanomedicines in the blood cannot escape from vascular confinement and are not readily able to extravasate across the endothelium. Moreover, neither their extravasation in areas of high vascular permeability, nor their accumulation in the vicinity of target cells, signify they are bioavailable unless the drug is released or the nanomedicine is endocytosed. These factors make it difficult to determine the pharmacokinetics and biodistribution of nanomedicines. (iii) In the blood circulation, nanomedicines tend to aggregate, adsorb plasma proteins, and prematurely release their cargos; also, they are phagocytosed by circulating monocytes or tissue macrophages to be degraded. This gives rise to the emergence of new modes of toxicity, including hemolysis, inflammation, oxidative stress, and impaired lysosomal or mitochondrial function. In the case of BNZ, it is important to note that the potential toxicity of oral BNZ-loaded nanomedicines would result from absorbed free BNZ. The toxicity of intravenous nanomedicines (e.g., pH-sensitive liposomes or polymersomes) instead, would be caused by the interaction of the nanomedicine structure with blood components, and its nature may completely differ from that raised of free BNZ (or other 2-nitroimidazole compounds). (iv) Potential acute toxicity of intravenous nanomedicines cannot be observed in rodents, which are not suitable predictive models for potentially lethal reactions such as the complement activation-related pseudo-allergy effect [122]. Investigation of chronic toxicity is time-consuming, and analyzing chronic toxicity data is more demanding [123].

**Complex regulatory aspects:** The regulatory decisions on nanomedicines can only be made based on individual assessments of benefits and risks [123]. The lack of universal regulatory protocols for good manufacturing practices of nanomedicines makes their quality control aspects overly complex [110,124]. In addition, the regulatory framework for a given nanomedicine will change according to the country, thereby hindering approval and regulation [125–127].

**High cost:** The prohibitive costs of the raw materials, intensive research, and complicated production steps make nanomedicines expensive, discouraging the interest of pharmaceutical companies. For those reasons, the clinical therapeutic effect of nanomedicines must be much higher than that of conventional therapeutics [128–130]. Besides, the sophisticated nature of nanomedicines leads to complex issues related to patenting and the determination of intellectual properties [124].

### Status of current preclinical nanomedicines against CD and challenges ahead

**Figure 1:**
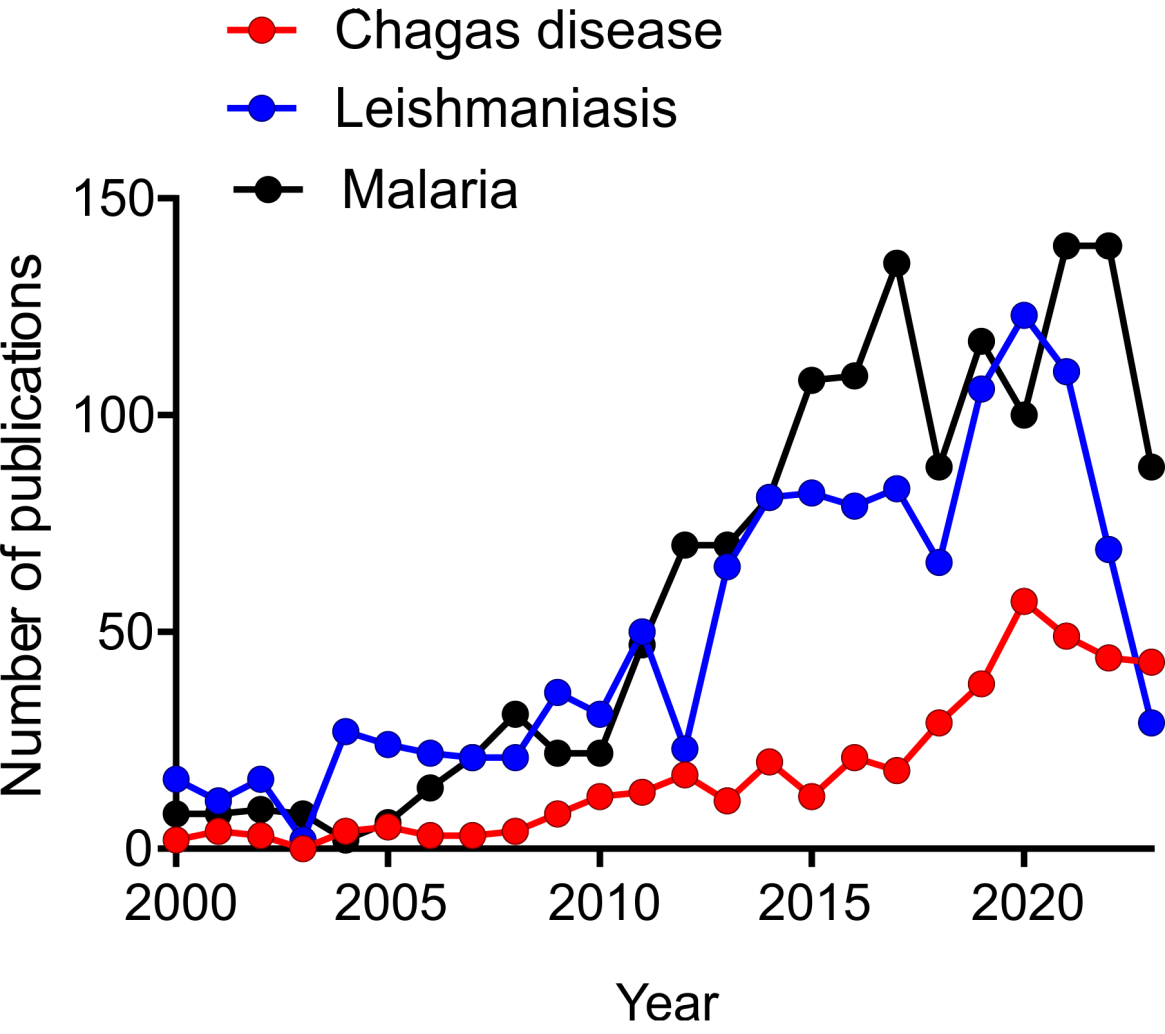
A search conducted on PubMed (17 November 2023), employing the following keywords (articles make no distinction between treatment and diagnosis): “Chagas and liposomes”, “Chagas and nanoparticles”, “Chagas and micelles”, “cruzi and liposomes”, “cruzi and nanoparticles”, “cruzi and micelles”; “leishmania and liposomes”, “leishmania and nanoparticles”, “leishmania and micelles”; “malaria and liposomes”, “malaria and nanoparticles”, “malaria and micelles”, “plasmodium and liposomes”, “plasmodium and nanoparticles”, and “plasmodium and micelles”.

While the reports published over the last 20 years on treatments against CD and leishmaniasis are comparable in number, those against malaria (a parasitosis endemic to Asian countries) are more than fourfold higher. According to a search that includes diagnosis and treatment (Figure 1), the reports on anti-CD nanomedicines in the last 20 years are no more than a few hundred. This means that despite its high burden for developing countries, CD is neglected not only by pharmaceutical companies and governments but also by the academic [131] community, including nanomedical researchers.

The failure of the only large-scale multinational project aiming for cheap nanomedicines against CD was a missed unique opportunity since the original idea of bypassing liver metabolism was reasonable for a toxic drug such as BNZ. This project, gathering pharmaceutical companies, state institutions, and scientists, was sufficiently funded to lay the groundwork for a potential future commercial product. Seeking to increase the solubility of BNZ is a reasonable strategy if BNZ is considered a class-II drug. In this regard, most contemporaneous *in vitro* reports on BNZ-based nanomedicines aimed to increase BNZ solubility dismissed the fact that oral nanomedicines are not absorbed into the blood circulation. Since the recently discovered non-replicative state of parasites seems to be less susceptible to BNZ-induced damage, *in vitro* models of dormant parasites may be useful to explore the effect of endocytosed nanomedicines on these forms. The results would be suitable, however, only for intravenous nanomedicines. The much scarcer reports on the *in vivo* activity of oral BNZ-based nanomedicines, show trypanocide effects on acute/chronic and even immunocompromised mouse models, suggesting a cure. In these studies, the plasma levels of BNZ remain to be determined. This is important given, for example, the significant increase in the drug’s bioavailability provided by nanocrystals, and the potential toxic effects observed in humans, which some authors have linked to high BNZ plasma levels. While BNZ-based nanomedicines such as BNZ-NC are advantageous because of the extraordinarily high BNZ loading, unexplored issues may arise from its huge specific surface area and interfacial free energy. Nanocrystals are thermodynamically unstable, with a tendency to aggregate, undergoing Oswald ripening and changes in the crystal polymorph, inducing potential tissue irritation [132–133]. Also, if ADRs were associated with an intrinsic susceptibility of patients, reducing BNZ plasma levels would not be helpful. Hence, what type of nanomedicines could solve the problem of BNZ-based medication? Targeting BNZ to diseased tissues while avoiding healthy ones, for instance, could alleviate the problem. However, for controlled biodistribution, nanomedicines must be intravenously administered. This fact, as discussed previously, raises the complexity of the therapeutic strategy to limits that may impair its implementation in developing countries. Unfortunately, early attempts to treat CD with intravenously administered nanomedicines failed or were discontinued.

The assessment of pharmacokinetics and biodistribution of nanomedicines is complicated since many aspects of human disease cannot be modeled in animals [134]. With respect to standardized models needed for predictive preclinical evaluation of novel therapies against CD [135], issues regarding assay design and endpoint definitions persist [136]. Moreover, vessel diameters, irregular permeability, and microenvironments of diseased tissues from animal models are hardly comparable to those of healthy or diseased humans. This constitutes a problem for the development of predictive preclinical models. In addition, alternatives such as “Replacing, Reducing, and Refining” the use of animals in research (3R concept) [137] toward the more predictive and less cruel use of 3D human cell cultures and microfluidics, are still too costly for routine research in developing countries [138–139]. The therapeutic performance of intravenous nanomedicines strongly depends on the anatomical pathological context, mainly on the extent of inflammation or retention permeation effect, and the extracellular matrix tightness of target tissues. This aspect, practically overlooked in animal models of Chagas disease, could distort the biodistribution of nanomedicines, yielding falsely promising preclinical results, as occurred with antitumor nanomedicines administered to xenografts tumor models [140].

Finally, to successfully develop nanomedicines, disease-led rather than formulation-led design is essential [141]. In treatments performed with intravenous nanomedicines, hydrodynamic diameter (much larger than that of a low-molecular-weight drug) and surface nature, are critical to maximize the access to target sites. The extracellular or intracellular character of the targets must be known beforehand, as well as the diseased tissue’s location, the presence of acidity, oxidative stress, or associated inflammation. The pathophysiology and the nature of targets in CD, however, seem not to be completely defined yet. Our limited understanding of the infection process, pathology progress, and its long-term nature makes it difficult to find new drugs for better treatments, or vaccines to prevent or treat CD [142]. It has been suggested, for example, that the classical view of the infective cycle is superficial and that the process in mammalian hosts is certainly more complex. Acceptable formulations for the treatment of the chronic phase [136], for example, should reach intracellular targets. But what is the nature of these targets and how feasible for nanomedicines is it to access them? Recently, the presence of intracellular epimastigote-like forms, named zoids (cells with kinetoplast but no nucleus, which quickly die and are degraded by the host cell) and of metabolically quiescent or dormant amastigotes, have been described. Dormant amastigotes can reside over long periods of time in chronically infected tissues and can spontaneously restart the infection, even after treatment, accounting for drug resistance in CD. An adaptive difference between *T. cruzi* strains to induce dormancy has also been suggested. Infected muscle or tissue macrophages can only be targeted by intravenous nanomedicines if local inflammation is manifested [39]. In the absence of inflammation, cells hosting dormant parasites could be inaccessible to nanomedicine extravasation. Determining the presence of inflammation is critical to predicting the success of intravenous nanomedicine-based treatment. Also, if BNZ is not effective against quiescent forms, a treatment with BNZ-based nanomedicines will not be of use to solve the problem.

The preclinical data gathered to date tells us that we are far from determining whether the efficacy and reduced toxicity of BNZ or 2-nitroimidazole-based nanomedicines would outperform the current oral BNZ-based treatment.

Drugs are not developed because of social or humanitarian reasons. The cost of drug development and the risks are high, and the times are long [143–144]. In the end, there must be certainty of making profit, and it is the pharmaceutical companies that take that risk. Investment in nanomedicines, thus, is of high risk and must yield a high reward. This magnification of cost deepens the challenge of developing drugs against endemic infections affecting millions of people in poor or developing countries. Even relatively high anticipated sales volumes may provide insufficient incentives if the expected pricing is low [145].

In this scenario, it can be predicted that *de novo* development of nanomedicines exclusively aimed at increasing bioavailability, reducing toxicity, or for targeted delivery of drugs such as the relatively well-known BNZ is not an impossible, but an expensive, enterprise. Much more expensive and riskier will be developing nanomedicines for the delivery of non-approved molecules.

In 2021, the effect of a subcutaneous immunostimulant with imiquimod loaded in nanoarchaeosomes (a type of lipid vesicles, nanoarc-imq) on an acute model of CD was reported. The lipids from nanoarc-imq showed extraordinary resistance to factors that normally reduce the structural stability of nanoparticulate material. The treatment not only reduced parasitemia but also eliminated inflammation and cardiac fibrosis more efficiently than BNZ [146]. These preliminary results, based on immunostimulation in the absence of antigens, matched those achieved with intravenous administration of liposomal amphotericin B in acute mice models of CD. Structurally simple formulations like that made of lipid nanovesicles, the same as nanocrystals, are examples of nanomedicines that, because of their easy industrial scale-up, structural assessment, and cheaper production (compared to that required for drug delivery or vaccination), could make it to the market against CD. Consider the development of Mosquirix™, which took nearly 35 years, for instance. In comparison, the factors that made the rapid appearance of nanomedical anti-infective vaccines possible were long-term investments in research infrastructure and major government interventions, which absorbed much of the risk from research and development. None of these elements exist in most countries where CD is endemic. Repurposing nanomedicines already available, as happened with liposomal amphotericin B and leishmaniasis could be a possibility. However, today there are no prospects in sight for nanomedicines with potential anti-CD activity on the market.

## Conclusion

Overall, the main difficulty for developing anti-CD nanomedicines would not lie in the complexity of the pathology itself, but in the neglected character of CD [147]. The challenge of improving the treatment of the chronic phase of CD has been posed for decades, and the classic pharmaceutical industry has not been able or interested to face it. Will nanomedicine be able to do it? The answer is more political than technical.

## Data Availability

Data sharing is not applicable as no new data was generated or analyzed in this study.
